# Role of NKCC1 and KCC2 in Epilepsy: From Expression to Function

**DOI:** 10.3389/fneur.2019.01407

**Published:** 2020-01-17

**Authors:** Ru Liu, Junling Wang, Shuli Liang, Guojun Zhang, Xiaofeng Yang

**Affiliations:** ^1^Neuroelectrophysiological Laboratory, Xuanwu Hospital, Capital Medical University, Beijing, China; ^2^Center of Epilepsy, Center for Brain Disorders Research, Capital Medical University, Beijing, China; ^3^Center of Epilepsy, Beijing Institute of Brain Disorders, Beijing, China; ^4^Guangzhou Regenerative Medicine and Health Guangdong Laboratory, Guangzhou, China; ^5^Department of Functional Neurosurgery, Beijing Children's Hospital, Capital Medical University, Beijing, China; ^6^Department of Functional Neurosurgery, Xuanwu Hospital, Capital Medical University, Beijing, China

**Keywords:** epilepsy, NKCC1, KCC2, electrophysiology, bumetanide

## Abstract

As a main inhibitory neurotransmitter in the central nervous system, γ-aminobutyric acid (GABA) activates chloride-permeable GABAa receptors (GABAa Rs) and induces chloride ion (Cl^−^) flow, which relies on the intracellular chloride concentration ([Cl^−^]_i_) of the postsynaptic neuron. The Na-K-2Cl cotransporter isoform 1 (NKCC1) and the K-Cl cotransporter isoform 2 (KCC2) are two main cation-chloride cotransporters (CCCs) that have been implicated in human epilepsy. NKCC1 and KCC2 reset [Cl^−^]_i_ by accumulating and extruding Cl^−^, respectively. Previous studies have shown that the profile of NKCC1 and KCC2 in neonatal neurons may reappear in mature neurons under some pathophysiological conditions, such as epilepsy. Although increasing studies focusing on the expression of NKCC1 and KCC2 have suggested that impaired chloride plasticity may be closely related to epilepsy, additional neuroelectrophysiological research aimed at studying the functions of NKCC1 and KCC2 are needed to understand the exact mechanism by which they induce epileptogenesis. In this review, we aim to briefly summarize the current researches surrounding the expression and function of NKCC1 and KCC2 in epileptogenesis and its implications on the treatment of epilepsy. We will also explore the potential for NKCC1 and KCC2 to be therapeutic targets for the development of novel antiepileptic drugs.

## Introduction

Many factors have been shown to play important roles in facilitating the development of epilepsy in humans and animal disease models, including genetic mutations, environmental factors during development, and brain injury (1–3). Although the exact mechanism of epileptogenesis is not yet fully understood, epilepsy is widely believed to be associated with an excitatory-inhibitory imbalance (4, 5). There is increasing evidence in recent literature to suggest that epileptogenesis results primarily from a deficit of GABA inhibition (6, 7).

Seventy to eighty percent of neocortical neurons are excitatory glutamatergic pyramidal neurons while the remaining are mostly GABAergic inhibitory interneurons with diverse characteristics (8, 9). Based on experimental studies involving seizure models and epilepsy patients, we know that the down-regulation of GABA receptor subunits, loss of GABAergic interneurons, and decreased frequency and amplitude of spontaneous synaptic GABA currents from pyramidal neurons contribute to decreased GABAergic inhibition (7, 10–12). In neurons, NKCC1 and KCC2 serve as two main regulators of GABA receptors' function by maintaining [Cl^−^]_i_ homeostasis (13). A disruption in the balance between NKCC1 and KCC2 induced by unknown causes may result in ineffective GABA inhibition (14, 15). This suggests a more complex scope of functions for GABAergic signaling than just global inhibition.

Approximately 30% of patients with epilepsy fail to respond to current therapies, so there is increasing demand for novel antiepileptic drugs (AEDs) and other therapeutic strategies (16, 17). Current AEDs enhance GABAergic function (18), either by modulating the post-synaptic GABAa Rs (barbiturates and benzodiazepines) or by acting on synaptic GABA availability (vigabatrin). The challenge faced by physicians is that conventional AEDs approved for use in adults have poor effects in neonates and may actually potentiate seizures in this patient population (19–22). In this review, we introduce the main chloride transporters associated with GABA signaling and discuss their regulatory dynamics on GABA functions at various levels ranging from genotypes to functional alterations and phenotypes. We will also discuss the possible antiepileptic effect of the NKCC1 blocker, bumetanide, as a promising AED.

## The Role Of Cation-Chloride Cotransporters In Chloride Homestasis In Neural Cells

Intracellular chloride concentration is regulated by cation-chloride cotransporters in neural cells and it changes during development. The function of GABA may vary between different developmental stages (23).

### Physiological Effects of Cation-Chloride Cotransporters in the Brain

There are nine kinds of glycoproteins in the cation-chloride cotransporter (CCC) family. They each have apparent molecular weights of 120–200 kDa and are encoded by Slc12a1-9 genes (24). Seven of the nine are plasmalemmal ion transporters, which are divided into three categories: two NKCCs (NKCC1 and NKCC2), one Na-Cl cotransporter (NCC), and four KCCs (KCC1-4) (24, 25). All CCCs except for NKCC2 and NCC are found in the brain and each is expressed in specific cell types, brain regions, or developmental stages (26, 27). NKCC1 and KCC2 are two main secondary active transporters which work by accumulating and extruding Cl^−^, respectively.

KCC2 is encoded by Slc12a5 and is exclusively expressed in the plasma membrane of somata and dendrites on pyramidal neurons and interneurons from the hippocampus and neocortex. It pumps Cl^−^ across the plasma membrane out of the cell. In contrast, NKCC1, which is widely expressed in central and peripheral neurons as well as glial cells, is encoded by SLC12a2 and facilitates uptake of Cl^−^ into cells (13, 28). KCC2 is composed of two splice variants: KCC2a and KCC2b. KCC2b is responsible for establishing hyperpolarizing GABAa receptor-mediated transmission (29). NKCC1a and NKCC1b are two highly homologous NKCC1 isoforms. The NKCC1a isoform exhibits mRNA expression primarily in the brain (30). NKCC1 and KCC2 are comprised of 12 membrane-spanning segments, 6 extracellular loops, and intracellular N- and C-terminals. They differ in the position of regulatory sequences, phosphorylation sites, and long extracellular loops (25, 31, 32). In immature neurons, an age-specific upregulation of NKCC1 and a relative deficiency in KCC2 loads more Cl^−^ into the cell, resulting in a net Cl^−^ outflow and subsequent depolarization when GABA activates GABAa Rs. Conversely, higher KCC2 and less NKCC1 expression results in a net Cl^−^ influx in adult neurons (33–35). The developmental shift in expression and/or function of NKCC1 and KCC2 has sparked a large number of studies to understand the physiological and pathological mechanisms of this inreverse function in GABA.

### The Profiles in NKCC1 and KCC2 During Development

There are a number of studies stating that downregulated NKCC1 and upregulated KCC2 lead to a switch in equilibrium potential of chloride (${\text{E}}_{{\text{Cl}}^{-}}$) from depolarizing to hyperpolarizing during the neonatal period in “altricial” species (23, 36–38). Dzhala found that [Cl^−^]_i_ was maintained at low levels in adult neurons with high KCC2 and low NKCC1 expression at postnatal day 21 (P21) in rodents. In contrast, immature neurons accumulated Cl^−^, leading to membrane depolarization at postnatal day 5 (P5) (34, 39). However, quite different from what is observed in rodents, KCC2 mRNA is already present in abundant amounts at embryonic day 42 (E42) in guinea pigs (40). Notably, similar results was got in the perinatal human hippocampus and neocortex (34, 41). Recent research has shown that the expression of NKCC1 and KCC2 may occur outside the aforementioned developmental windows (42). There are compelling differences in epilepsy subtypes between the sexes (43), and the developmental expression of KCC2 and NKCC1 also exhibit sexual dimorphism (44–46). The expression profiles of NKCC1 in male rats have previously been reported by Dzhala (34, 46). There is a peak in the relative expression of NKCC1 observed in males but not in females at P9 and P11 separately in the hippocampus and entorhinal cortex. The expression of KCC2 was significantly higher in females than in males, particularly at P1 and P9-15 both in the hippocampus and entorhinal cortex (46). This is also consistent with more depolarizing GABAergic signaling previously reported in P4-14 male rats compared to female rats (45). In addition, in terms of CCCs, levels of transcription and protein expression may not be consistent with expected [Cl^−^]_i_. However, it should not be ignored that inward and outward transport rates and changes of post-transcription related modification, such as protein phosphorylation, participate in chloride homeostasis (47, 48).

### The Role of Chloride Concentration Homeostasis in GABAergic Excitability

**Figure 1:**
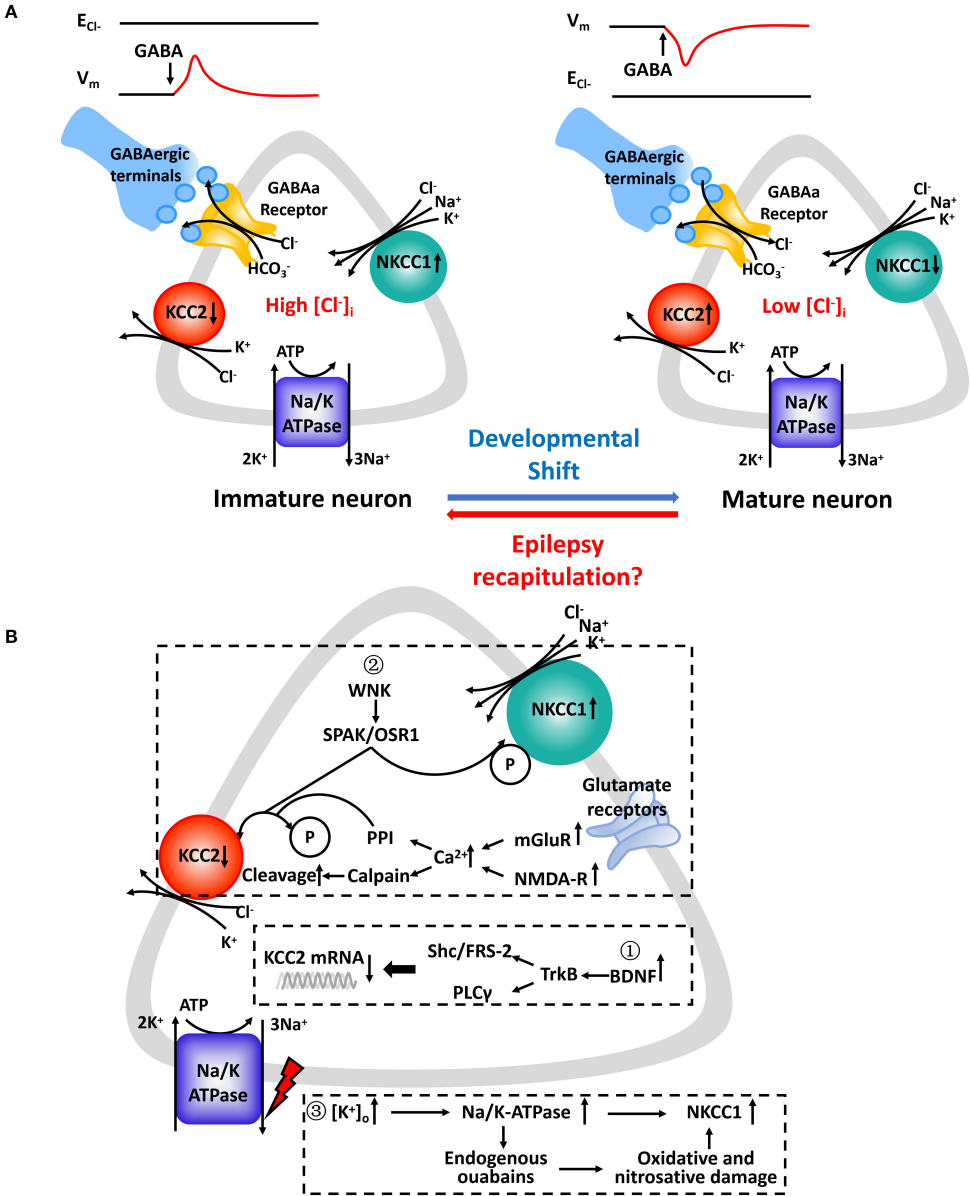
**(A)** Chloride concentration regulatory mechanisms underlying GABAa receptor-mediated responses in immature and mature CNS neurons. (Left): Upregulated NKCC1 is the main regulator which mediates Cl^−^ uptake in immature CNS neurons in parallel with downregulated KCC2 or in its absence. (Right): KCC2 is the principal K-Cl cotransporter in parallel with subsequent downregulated NKCC1 in mature CNS neurons. Cl^−^ transported by NKCC1 and KCC2 is facilitated by an electrochemical gradient of Na^+^ and K^+^ created by the Na^+^/K^+^-ATPase. The relative activity of NKCC1 and KCC2 and their opposing effects on [Cl^−^]_i_ determines the value of E_Cl−_ relative to the membrane potential (V_m_). GABA binds to corresponding GABAa receptors and opens ligand-gated Cl^−^ channels which are also permeable to HCO_3_^−^. Expression profiles of NKCC1 and KCC2 varies at different developmental stages. In several pathophysiologic conditions, such as epilepsy, neurons experience “recapitulation” and dedifferentiation to some key and special stages of early neuronal development. **(B)** Molecular cascades leading to expressive and functional abnormalities in NKCC1 and KCC2. Expression levels of NKCC1 and KCC2 mRNA ①, protein levels ②, and designated regulatory pathways ③ have been shown to be altered. Summarized findings are from animal models as well as human studies.

GABA, working as a major inhibitory transmitter of the mature central nervous system, is important in controlling neuron excitability and maturation and forming emerging networks. It binds to GABAa receptors, which are ligand-gated chloride channels, and triggers subsequent conformational changes (49). Then, the complex facilitates the passive flow of Cl^−^ depending on the neuron's E_cl_^−^ (50). In immature neurons, E_cl_^−^, which is affected significantly by minor changes in [Cl^−^]_i_, becomes positive relative to the resting membrane potential (V_m_) when [Cl^−^]_i_ is relatively high. However, ${\text{E}}_{\text{Cl}}^{-}$ is more negative than V_m_ when [Cl^−^]_i_ is low and the activation of GABAa Rs triggers Cl^−^ influx (Figure 1A).

It is a major misconception that depolarizing GABA actions mean excitation and that a more negative equilibrium potential for GABA (E_GABA_) is a prerequisite for inhibitory GABAergic function. First, because of intrinsic outward rectification of GABAa currents, inactivation of Na^+^ channels and activation of K^+^ channels caused by depolarizing GABA action may lead to more hyperlarizing GABAergic action (24). Second, depolarized GABA affects targeted cells via its shunting action. Postsynaptic activation of GABAa Rs leads to decreasing membrane resistance, increasing background conductance, and reduced efficacy of excitatory signals in space and in time. In other words, it may lead to a more effective inhibitory action by reducing the action potential threshold (51–53). Third, when GABA binds to GABAa Rs, bicarbonate (HCO_3_^−^) flows through GABAa Rs to a lesser extent compared to Cl^−^ (54). A net efflux of HCO_3_^−^ depolarizes E_GABA_ and may even control the polarity of GABAergic responses (55). It has been reported that HCO_3_^−^ generated by the catalytic activity of carbonic anhydrase not only paricipates in the regulation of neuronal volume and pH but also contributes to chloride homeostasis by affecting the Na^+^-driven Cl^−^/bicarbonate exchanger and the Cl^−^/HCO_3_^−^ exchanger AE3 (56, 57). However, due to the ~5-fold increase in permeability of Cl^−^ over HCO_3_^−^, E_GABA_ lies closer to the E_cl_^−^ than E_HCO3_^−^ in most neurons. Thus, [Cl^−^]_i_ is still a critical determinant in fast-synaptic inhibition (12, 53). It had been reported that depolarization by GABA controls early network activity in the developing hippocampus and neocortex (58, 59). During development, the activation of GABAa Rs leads to neuron depolarization and activation of Ca^2+^ signals in immature cells (60). GABAa receptor-mediated depolarization appears to play a decisive role in early network activities (58), circuit formation (61, 62), neuronal migration (63), and synapse maturation (64).

## Dysregulation Of NKCC1/KCC2 IN Epilepsy

### Changes in Function and Expression of NKCC1 and KCC2 on Animal Seizure Models

Clinical studies have shown that children are more likely to have epileptic seizures than adults, and the neonatal period in particular has the greatest incidence of seizures (1.8–3.5%) (65, 66). There is evidence that GABA has a depolarizing effect in the healthy newborn rat cortex (67). During the postnatal period, an increased expression of KCC2 shifts the polarity of GABAergic responses from depolarizing to hyperpolarizing in the mature neuron (34, 40). Researchers observed that elevated [Cl^−^]_i_ caused pathologic neuronal and network hyperexcitability in animal models, including knockout mice carrying a genetic loss of NKCC1 or KCC2 (1, 34, 68, 69). Wang et al. described the expression of NKCC1 and KCC2 based on electrophysiological studies of patients with epilepsy (12). Bumetanide, a specific inhibitor of NKCC1, may be a powerful tool to test whether NKCC1 is responsible for the accumulation [Cl^−^]_i_ (34).

In different animal models of chronic epilepsy, a significant upregulation of NKCC1 and downregulation of KCC2, both in mRNA and protein levels, have been reported (12). Depolarized E_GABA_ and accumulation of [Cl^−^]_i_ were consistent with the corresponding increased expression of NKCC1 and decreased expression of KCC2. However, studies on E_GABA_ shifts have reported inconsistent findings in animal models of epilepsy and are currently controversial (Table 1).

**Table 1:** Changes in function in experimental neonatal epilepsy models.

**References**	**Experimental epilepsy models**	**Animals**	**Brain regions**	**Changes**
**CONSISTENT WITH HIGH NKCC1 AND LOW KCC2 EXPRESSION**				
***In vivo***				
Dzhala et al. (34)	KA-induced seizures	P9-12 rats	Hippocampus	Bumetanide attenuated electrographic seizure in neonatal rats.
Mares (70)	PTZ-induced seizures	P7,12,18 male albino rats of Wistar strain	–	Bumetanide only decreased tonic phase of P12 rats which showed higher NKCC1 expression than other periods.
Mazarati et al. (71)	Kindling-induced seizures	P11, P14, P21 Wistar rats	Hippocampus	Bumetanide only increased ADT and shortened ADD in P11. Bumetanide delayed the occurrence, and reduced the number of full motor seizures.
Koyama et al. (67)	Hyperthermic seizure model	P11 wildtype or transgenic SD male rats	Hippocampus	Bumetanide rescued granule cell ectopia and blocked increased susceptibility to adult seizures after febrile seizures at P11.
***In vitro***				
Dzhala et al. (34)	Bath application of high [K^+^]_o_	P7-23 rats	Hippocampal slice	Bumetanide suppressed and depressed IED, ID in P7-9 and P10-12 rats, respectively.
		P7-9 NKCC1^−/−^ mice	Hippocampal slice	E_GABA_ was more hyperpolarized and bumetanide did not change the amplitude and duration of IEDs.
Dzhala et al. (68)	Bath application continuous 5 h of low-Mg^2+^ solution	P4-7 rats	Intact hippo- campal formations	Bumetanide had a strong action in controlling ISIs and hyperpolarize E_GABA_.
Rheims et al. (72)	Bath application of 4-AP	P6-P9 rats	Neocortex slice	Bumetanide inhibited generation of IISs and prevented their trans- formation to ISs.
**INCONSISTENT WITH HIGH NKCC1 AND LOW KCC2 EXPRESSION**				
***In vitro***				
Kilb et al. (73)	High [K^+^]_o_ model Disinhibition model Low-Mg^2+^ model Kainate model 4-AP/low-Mg^2+^ model	P4-7 rats	Whole hippo- campus	Bumetanide could attenuate epileptiform activity. Bumetanide had only little effect on interictal discharges. Bumetanide did not attenuate epileptiform activity but instead enhanced interictal activity. Bumetanide enhanced epileptiform activity. Bumetanide attenuated LRD like epileptiform activity.
Zhu et al. (74)	Bath application of 4-AP	P9-P13 NKCC1^+/+^ and NKCC1^−/−^ mice	Hippocampaus	Bumetanide aggravated 4-AP induced seizures and enhanced neuronal excitability.
Nardou et al. (75)	Bath application of Kainate	P7-8 Wistar rats	Interconnected intact hippo-campus	Bumetanide did not prevent generation and propagation to the contralateral hippo-campal and the formation of an acute epileptogenic mirror focus.
Khirug et al. (76)	Kainate induced and bath application of kainate	P5-7 and P16 Wistar male rat pups	Hippocampus	Enhanced KCC2 function and a consequent negative shift in E_GABA_ followed by seizure activity.
Awad et al. (77)	Freeze lesion and hyperthermic seizures	P18-22 or P11-15 male rats	Hippocampus	Amplitude of mEPSC in CA1 pyramidal neurons reduced significantly and E_GABA_ increased which related to KCC2 over-expression.

*4-AP, 4-aminopyridine; ADT, after discharge threshold; ADD, after discharge duration; ID, ictal discharge; IED, interictal epileptic discharges; IISs, interictal seizures; ISs, ictal seizures; ISI, inter seizure intervals; KA, kainae; PTZ, pentylenetetrazol; mEPSC, miniature excitatory postsynaptic current; SD, Sprague-Dawley*.

Dzhala found that bumetanide contributed to more hyperpolarized E_GABA_. He depressed synchronous bursts of action potentials by combining local field potential and whole-cell recordings of epileptiform activity induced by 8.5 mM [K^+^]_o_ and illustrated that NKCC1 facilitates seizures in the developing brain (34). Genetic loss of KCC2 in diverse model organisms including worms, flies, fish, and mice resulted in less Cl^−^ extrusion, more increase in [Cl^−^]_i_, and pathologic neuronal and network hyperexcitability (78). Dysregulation of NKCC1 and KCC2 has been shown to occur in various conditions, such as hyperthermic seizure, hypoxic-ischemic encephalopathy, and sevoflurane- induced seizures (67, 79, 80). However, there are still differing opinions on the effects of altered NKCC1 and KCC2 on epileptic activity. For example, a different point proposed by Zhu et al. illustrated that NKCC1 acts as an anticonvulsant by inhibiting the generation of seizures instead of facilitating seizure activity (74). Another view is that increased KCC2 protein levels are accompanied by a depolarized E_GABA_ in cortical dysplasia and hyperthermia-induced seizures, suggesting that KCC2 overexpression may increase febrile seizure susceptibility (77). In contrast to downregulation of KCC2 in seizure models, several scholars have shown that a single seizure episode induced by kainate injection of P5-7 in rats can increase the Cl^−^ extrusion capacity of rat hippocampal CA1 neurons and increase the activity-dependent regulation of KCC2 function (76). In addition to understand the regulation of NKCC1 and KCC2 in various neonatal seizure and epilepsy models, it is worth exploring changes in NKCC1 and KCC2 in mature neurons (Table 2).

**Table 2:** Changes in function of NKCC1 in experimental adult epilepsy models.

**References**	**Experimental epilepsy models**	**Animals**	**Brain regions**	**Findings**
**CONSISTENT WITH HIGH NKCC1 AND LOW KCC2 EXPRESSION**				
***In vivo***				
Robel et al. (81)	Glioma-induced epilepsy	Adult genetically induced mice	Cortex	Bumetanide eliminated seizure in a subset of glioma-induced seizure rats.
Sivakumaran and Maguire (82)	KA-induced seizure	Adult male C57BL/6 mice	Hippocampus	Bumetanide decreased KA-induced ictal activity.
Wang et al. (83)	TBI-induced seizure	Adult male C57bc/6j mice and adult NKCC1^−/−^ mice	Cortex	NKCC1 upregulation may contribute to increased seizure susceptibility, which occurs after TBI.
***In vitro***				
Pathak et al. (84)	Pilocarpine induced chronic seizures	Male SD rats	Hippocampus	Depolarized E_GABA_ and reduced KCC2 functioned in granule cells of DG.
Bragin et al. (85)	Li low-dose pilocarpine induced seizures	Adult Wistar rats	Cortex and hippocampus	Depolarizing shift of the inhibitory postsynaptic potential reversal potential in layer 5 neurons caused by upregulation of NKCC1 and downregulation of the KCC2.
Barmashenko et al. (86)	Pilocarpine-induced seizures	P30 male Wistar rats	Hippocampus	E_GABA_ was significantly more positive in all regions and increased function of NKCC1 contributed to hyperexcitability.
Robel et al. (81)	Glioma-induced seizures	Adult genetically induced mice	Cortex	Neuronal hyperexcitability was related to increased expression of NKCC1 and decreased KCC2.
Sivakumaran and Maguire (82)	0-Mg^2+^-induced seizure-like events	Adult male C57BL/6 mice	Hippocampus	Bumetanide decreased 0-Mg^2+^-induced seizure-like events.
Kourdougli et al. (87)	Pilocarpine hydrochloride induced seizures	Adult male Wistar rats	Hippocampus	Bumetanide restored NKCC1 and KCC2 expression and rewired glutamatergic neuronal circuits within the dentate gyrus.
Santos et al. (88)	Long-term alcohol exposure induced NEA	Adult male Wistar rats	Hippocampus	Enhanced NKCC1 contributed to increasing DC shifts and bumetanide suppressed the DC shift increase and diminished NEA.
Wang et al. (83)	TBI-induced seizures	Adult male C57bc/6j mice and adult NKCC1^−/−^ mice	Cortex	Enhanced NKCC1 mediated cortical hyperexcitability.
**INCONSISTENT WITH HIGH NKCC1 AND LOW KCC2 EXPRESSION**				
***In vitro***				
Viitanen et al. (89)	Bath application of isoguvacine	P15-35 rats	Hippocampus	KCC2 modulated Cl^−^ efflux contributing to GABAergic excitatory [K^+^]_o._
Hamidi and Avoli (90)	Bath application of 4-AP	Adult male SD rats	Cortex	Enhanced KCC2 activity facilitated ictogenesis.

*DG, dentate gyrus; HFS, high-frequency stimulation; NEA, nonsynaptic epileptiform activities; SD, Sprague-Dawley; SE, status seizure; TBI, traumatic brain injury*.

Several studies have shown that enhanced NKCC1 and decreased KCC2 function is consistent with increased NKCC1 and decreased KCC2 mRNA and protein levels in different adult animal seizure models. The phenomenon is named-“ontogenetic recapitulation,” which implies that neurons in the adult models had dedifferentiated to some key and special stage of early neuronal development. Bumetanide significantly decreases kainate-induced ictal activity *in vivo* and 0-Mg^2+^-induced seizure-like events *in vitro*, which implies that impaired chloride plasticity may contribute to in seizure progression (82). It is hypothesized that an abnormal depolarizing GABAergic function caused by an imbalance in NKCC1 and KCC2, may cause rewiring of excitatory glutamatergic neuronal circuits and play a role in the epileptogenic processes (87). NKCC1 not only plays a key role in facilitating synaptic epileptiform activity, but also in non-synaptic epileptiform activity in the dentate gyrus (91). Increased seizure susceptibility and depolarized E_GABA_ caused by chronic stress may imply an imbalance in chloride homeostasis and can be reversed with bumetanide (92). KCC2 drives Cl^−^ out of the neuron against its electrochemical driving force through the “Cl^−^ pump” mechanism and is postulated to be the force for mediating Cl^−^ outflow in mammalian cortical neurons (93). Moreover, significantly decreased KCC2 protein and mRNA levels with an impaired chloride clearance causes a positive shift in E_GABA_, resulting in decreased inhibition in the dentate gyrus. This functions as a filter impeding excessive or aberrant activity from propagating further into the circuit (94). The aforementioned studies in adult animal seizure models are controversial, but it is generally accepted that recurrent seizures and other traumatic insults may lead to downregulation of KCC2 and remodeling of NKCC1-dependent depolarizing GABAergic signaling (35). Hamidi detected high-frequency oscillatory events from field potential recordings and suggested that enhancing KCC2 activity may facilitate ictogenesis by increasing extracellular K^+^ (90). KCC2 expression returned to normal level and was even upregulated during the chronic phase, suggesting that a certain level of reorganization on seizure activity might be necessary for this increase (84).

The molecular cascades that lead to imbalanced CCCs and the possibility of progression from “activity-dependent” chloride plasticity to “pathologic” chloride plasticity remain largely unknown (12). As studies stand presently, there are some factors that lead to an upregulation of NKCC1 and downregulation of KCC2 in neurons in epilepsy (Figure 1B). First, the brain-derived neurotrophic factor (BDNF) and tyrosine kinase receptor B (TrkB) signaling pathways endogenously release more BDNF, which binds to TrkB receptors and activate src homology domains that contain transforming protein/FGF receptor substrate 2 (Shc/FRS-2) and phospholipase Cγ (PLC-γ). This has been shown to reduce the expression of KCC2 mRNA in various animal models of epilepsy (79, 95, 96). Second, different phosphorylation sites play different roles in the surface stability and activity of KCC2. The phosphorylation of residue serine-940 (S940) within the KCC2 cytoplasmic domain leads to increased KCC2 activity as well as its increased accumulation on the plasma membrane (97). Ca^2+^-dependent mechanisms initiated by the accumulation Ca^2+^ from metabotropic glutamate receptors (mGluRs) and glutamatergic NMDA receptors lead to downregulation of KCC2. It also coincides with protein phosphatase 1 dependent dephosphorylation of S940 and activation of calcium-activated protease calpain, which is involved in the cleavage of KCC2 (98, 99). Moreover, phosphorylation of Tyr 903/1087 of the KCC2 protein in pilocarpine-induced adult seizure mouse models leads to increased lysosomal degradation of KCC2 (100). Activation of with-no-lysine kinase (WNK), SPS1-related proline/alanine-rich kinases (SPAK), or SPAK homolog oxidative stress-responsive kinase1 (OSR1) pathways causes phosphorylation of threonin residues T906 and T1007, causing decreased KCC2 activity (47). The WNK-SPAK/OSR1 pathway also phosphorylates NKCC1 and promotes NKCC1 activity (101). Third, the role that Na^+^/K^+^-ATPase plays in epileptogenesis is not yet fully understood. The downregulation of ion transporters, such as plasmalemmal Na^+^/K^+^-ATPase, occurs via translocation of the protein and functional loss of transport activity and may reflect an adaptive response to an “energy crisis” (102, 103). Extracellular potassium uptake in neurons occurs via the Na^+^/K^+^-ATPase (104). Ion gradients created by the catalytic effect of Na^+^/K^+^-ATPase drives NKCC1 function by activating production of endogenous ouabains that cause oxidative and nitrosative damage (105). Reduced GABAergic potentials and enhanced coupling between excitatory postsynaptic potentials and spike firing are the primary mechanisms underlying the hyperexcitability associated with impaired Na^+^/K^+^-ATPase activity (106). Furthermore, molecular mechanisms related to the regulation of neuronal NKCC1 in epilepsy needs to be further explored.

Increasingly more studies have taken an interest in animal models that artificially and specifically lower the expression of ortholog KCC2. Genetic ablation of KCC2 resulted in increased seizure susceptibility (107), and may work through not only impaired Cl^−^ homeostasis but also dendritic spine dysfunction due to inability of KCC2 to bind to the cytoskeleton (32, 108). Transgenic technology made it possible to analyze the gene sequence for human SLC12a5, a mutant human gene in patients with seizure disorders (1, 109–112). This provided a potential target for electrophysiological studies aimed at exploring the pathogenesis of epileptic attacks. Unfortunately, while epileptiform discharges can be induced in animal seizure models, whether scientists can reproduce all aspects of human pathology in epilepsy remains uncertain (113).

### Changes of NKCC1 and KCC2 in Epilepsy Patients

Although there have been many acute and chronic animal models studies on altered NKCC1 and KCC2, there is still a need to perform studies on human epileptic brain tissue (114). Following are several studies investigating the changes of NKCC1 and KCC2 in epilepsy patients.

Dzhala investigated changes in NKCC1 and KCC2 levels in the human cortex and showed that NKCC1 level peaked from post-conceptional week (PCW) 31–41. KCC2 expression was ~2–25% of adult levels at PCW 31–41 and increased over the first year of life (34). Described as a specific malformation of cortical development, focal cortical dysplasia (FCD) is categorized into 3 main types. FCD type II is divided into two subtypes, FCD IIa and FCD IIb whose only difference histopathologically is the appearance of balloon cells (115). Lesions in FCD IIb have higher NKCC1: KCC2 ratios compared to lesions in FCD IIa. In other words, decreased GABAergic inhibition may represent a critical mechanism of epileptogenesis in FCD IIb compared to FCD IIa (11). In a recent study on pediatric FCD patients, interictal discharges sustained by GABAergic signaling were suppressed by blocking NKCC1. Meanwhile, perturbed Cl^−^ homeostasis related to decreased expression and changes in KCC2 have been observed in the same patients (7). Sharing similar pathological characteristics with FCD, dysplastic neurons in tuberous sclerosis complex (TSC) exhibit depolarizing GABAa Rs mediated spontaneous post-synaptic currents (PSCs), which are controlled by bumetanide (11). In general, a shift toward the excitatory effect of GABA may contribute to FCD and TSC epileptogenicity.

Based on results from several animal models of chronic epilepsy using kainate or pilocarpine, downregulation of KCC2 may play a critical role in the pathophysiology of temporal lobe epilepsy (TLE). The hippocampal subiculum serves as an origin for synchronous firing to spread to other regions of the temporal lobe during seizures. This region shows significant upregulation of NKCC1 mRNA and downregulation of KCC2 mRNA compared with the hippocampus proper or neocortex in drug-resistant mesial temporal lobe epilepsy (MTLE) (116, 117). Electrophysiology studies have shown that aberrant GABA excitation caused by altered NKCC1 and KCC2 contributes to the precipitation of epileptic seizures in TLE (117, 118).

What roles do different types of neurons play in the generation of epileptic activities? Depolarizing GABAa receptor-mediated PSCs in a minority of subiculum pyramidal cells have been shown to impair Cl^−^ homeostasis. Remodeling Cl^−^ homeostasis using bumetanide produced a hyperpolarizing shift in E_GABA_ and suppressed interictal activity. Among depolarized cells, most were immunonegative for KCC2, and all hyperpolarized cells in interictal events were immune-positive for KCC2 (119). In TLE, high-affinity glycine receptors (haGlyR), which are homologous to GABAa receptors, are increased in the hippocampus and this is inversely associated with a decrease in KCC2 expression (120). Contrary to what was mentioned previously, Karlocai et al. investigated differences in KCC2 expression in the hippocampus of TLE patients and control brain samples using immunocytochemistry and noted an overall increase in KCC2 expression in epilepsy patients (121). It has been disputed that the differences in KCC2 expression may result from differences in epileptic tissues, epileptic phases (i.e., acute, latent, chronic) (84), and brain regions studied (85, 119, 122, 123).

Analogous to FCD and TLE, brain tumors also exhibit a shift in GABAergic signaling from inhibitory to excitatory, which likely contribute to their epileptogenicity (124). Conti et al. studied peritumoral cortical tissues from epileptic patients afflicted by gliomas and found alterations in NKCC1 and KCC2 activity and a reduction in the hyperpolarizing effects of GABA, which likely contribute to epileptogenesis (124). Pallud et al. showed that in patients with gliomas, GABA depolarized 65% of pyramidal cells, which exhibited a perturbation of chloride homeostasis with a 144% increase in NKCC1 expression and a 42% decrease in KCC2 expression (125).

Human hypothalamic hamartomas associated with gelastic seizures is difficult to diagnose early in life and notoriously refractory to medical therapy (126, 127). Kim et al. expounded that altered expression of NKCC1 and KCC2 via GABAa receptor-mediated excitation contributes to epileptogenesis in hypothalamic hamartomas (128). In a recent study, a shift of E_GABA_ toward more depolarizing values and a parallel increase in the ratio of NKCC1 to KCC2 expression were found in Xenopus oocyte cell membranes obtained from brain tissues of Dravet syndrome patients (129). Differences in gene silencing also play a role as significantly lower NKCC1 DNA methylation and higher KCC2 DNA methylation has been shown in juvenile myoclonic epilepsy (130). However, we need to await further experiments based on human tissues to verify these altered patterns in brain disorders associated with epilepsy. Bumetanide, which rescues the accumulation of [Cl^−^]_i_ by suppressing NKCC1 specifically, reestablishes the hyperpolarizing GABA response and decreases epileptiform activity. Thus, it is a potential novel therapeutic avenue for the treatment of severe and intractable epilepsy.

## NKCC1/KCC2: Potential New Targets For Epilepsy Therapy?

Depolarizing responses mediated by GABAa Rs were first seen in hippocampal tissues obtained from human epileptic tissue (131). Although many adult epilepsy patients respond well to conventional AEDs—particularly those that potentiate GABAa Rs—these drugs are largely ineffective in controling neonatal epilepsy (68, 132). Consequently, it is critical that we pursue novel AEDs for epilepsy treatment.

Two widely used diuretic drugs have been implicated in the regulation of NKCC1 and KCC2: bumetanide which blocks NKCC1 and furosemide which targets several types of ion-transporters (133, 134). Furosemide, acts on multiple protein targets, such as KCC2, AE3, some subtypes of GABAa Rs, as well as carbonic anhydrase (135, 136). It exerts its anticonvulsant effect through a variety of complex mechanisms involving the regulation of [K^+^]_o_ and [Cl^−^]_i_ homeostasis, cell volume, and pH. Uwera et al. investigated the effects of furosemide on Cs-induced epileptiform activity and found that it worked by blocking neuronal KCC2 and AE3 transporters (134).

Kharod summarized the results of pre-clinical and clinical studies regarding model-specific efficacies of bumetanide (137). Here, we revisit these clinical studies briefly. Kahle et al. explored the clinical antiepileptic effects of bumetanide and found that bumetanide did not show any antiepileptic effects in the human neonate (138). Disappointingly, the first clinical phase I/II trial with bumetanide in 14 newborn infants with seizures showed that bumetanide was not effective enough as an adjunct to phenobarbital and increased the risk of hearing loss (139). Eftekhari et al. were the first to demonstrate the efficacy of bumetanide on reduction of seizure frequency in three adult patients with temporal lobe epilepsy (140). It was enlightening and inspiring to see decreased seizure frequency in all three patients and decreased epileptiform discharges in two of the three patients (140). A recent study of 30 patients with drug-resistant TLE showed that bumetanide can be an effective and relatively tolerable drug for patients with recalcitrant disease (141, 142). Of course, there are limitations and adverse side effects of bumetanide, which limit its application in clinical practice (35, 143). Therefore, it remains to be determined whether bumetanide will become a novel AED.

## Conclusion and Future Outlooks

NKCC1 and KCC2, two main members of the cell-type-specific and subcellular expression patterns of functional CCCs, regulate the effects of GABAergic signals by mediating steady-state [Cl^−^]_i_ gradients. Temporal shifts in GABAergic signaling have been identified during development. In early stages of embryonic development, depolarizing GABA activates synaptic receptors and regulates neuronal migration. Consistent with the excitatory effects of GABA, NKCC1, and KCC2 undergo similar changes in ontogenesis. However, in several pathophysiologic conditions, such as epilepsy, neurons revert to an immature state and demonstrate downregulation of KCC2 and upregulation of NKCC1.

We have summarized electrophysiology studies investigating the role of NKCC1 and KCC2 in animal epileptic models and *in vitro* studies using tissue from epilepsy patients. The most common conclusion of the role of these two cotransporters in epileptogenesis is that increased [Cl^−^]_i_, mediated by upregulation of NKCC1 and downregulation of KCC2, diminishes the efficacy of GABAergic inhibition or even reverts it to excitation. Changes in NKCC1 and KCC2 likely interact with multiple complex mechanisms and contribute to the pathogenesis of epilepsy. The diuretic bumetanide is widely used to inhibit neuronal NKCC1, particularly when its expression is abnormally increased in patients with epilepsy. However, there is still no convincing data confirming that bumetanide could be used in the clinic as an effective AED.

Several studies have shown that, at the cellular level, plasmalemmal channels and ion transporters are often multifunctional (144). Future experimental work on polymorphisms may provide additional insights into functional and dysfunctional CCCs. Further studies in human subjects are necessary to uncover the exact mechanism by which CCCs contribute to epileptogenesis.

## Author Contributions

RL, JW, SL, and GZ contributed to the conceptualization, designed the manuscript, and conducted literature searches. RL wrote the manuscript and made tables. XY and GZ revised the manuscript.

### Conflict of Interest

The authors declare that the research was conducted in the absence of any commercial or financial relationships that could be construed as a potential conflict of interest.
